# Current Evidence of Watermelon (*Citrullus lanatus*) Ingestion on Vascular Health: A Food Science and Technology Perspective

**DOI:** 10.3390/nu14142913

**Published:** 2022-07-15

**Authors:** Mônica Volino-Souza, Gustavo Vieira de Oliveira, Carlos Adam Conte-Junior, Arturo Figueroa, Thiago Silveira Alvares

**Affiliations:** 1 Nutrition and Exercise Metabolism Research Group, Multidisciplinary Center UFRJ-Macaé, Federal University of Rio de Janeiro, Macaé 27971-525, Brazil; mvolinosouza@gmail.com (M.V.-S.); gvo.vieira@gmail.com (G.V.d.O.); 2 Postgraduate Program in Food Science, Chemistry Institute, Federal University of Rio de Janeiro, Rio de Janeiro 21941-598, Brazil; carlosconte@hotmail.com; 3 Medical Science Institute, Multidisciplinary Center UFRJ-Macaé, Federal University of Rio de Janeiro, Macaé 27930-560, Brazil; 4 Department of Kinesiology and Sport Management, Texas Tech University, Lubbock, TX 79409, USA; 5 Food and Nutrition Institute, Multidisciplinary Center UFRJ-Macaé, Federal University of Rio de Janeiro, Macaé 27930-560, Brazil

**Keywords:** L-citrulline, endothelial function, arterial stiffness, blood pressure

## Abstract

The amino acid L-arginine is crucial for nitric oxide (NO) synthesis, an important molecule regulating vascular tone. Considering that vascular dysfunction precedes cardiovascular disease, supplementation with precursors of NO synthesis (e.g., L-arginine) is warranted. However, supplementation of L-citrulline is recommended instead of L-arginine since most L-arginine is catabolized during its course to the endothelium. Given that L-citrulline, found mainly in watermelon, can be converted to L-arginine, watermelon supplementation seems to be effective in increasing plasma L-arginine and improving vascular function. Nonetheless, there are divergent findings when investigating the effect of watermelon supplementation on vascular function, which may be explained by the L-citrulline dose in watermelon products. In some instances, offering a sufficient amount of L-citrulline can be impaired by the greater volume (>700 mL) of watermelon needed to reach a proper dose of L-citrulline. Thus, food technology can be applied to reduce the watermelon volume and make supplementation more convenient. Therefore, this narrative review aims to discuss the current evidence showing the effects of watermelon ingestion on vascular health parameters, exploring the critical relevance of food technology for acceptable L-citrulline content in these products. Watermelon-derived L-citrulline appears as a supplementation that can improve vascular function, including arterial stiffness and blood pressure. Applying food technologies to concentrate bioactive compounds in a reduced volume is warranted so that its ingestion can be more convenient, improving the adherence of those who want to ingest watermelon products daily.

## 1. Introduction

Several studies have demonstrated that watermelon (*Citrullus lanatus*) ingestion can increase nitric oxide (NO) bioavailability, an important vasoactive molecule that plays a critical role in the maintenance of vascular health [1,2]. Watermelon is a L-citrulline-rich source, and its ingestion can increase plasma levels of L-citrulline and L-arginine, an essential substrate for NO synthesis [3,4,5]. Thus, watermelon ingestion has been widely encouraged to improve vascular health in clinical populations.

Impaired vascular function is associated with reduced L-arginine availability and increased reactive oxygen species (ROS) that can negatively impact NO synthesis or increase its degradation [6,7]. For this reason, previous studies have investigated the effect of NO precursors-rich food on flow-mediated dilation (FMD, a gold standard measure of endothelial function), pulse wave velocity (a measure of arterial stiffness), and blood pressure in individuals with cardiovascular risk factors [2,8,9,10,11].

However, there is evidence reporting the absence of a positive vascular effect after watermelon ingestion [8,9,12]. Interestingly, the studies that failed to improve vascular parameters provided a low dose of L-citrulline in watermelon products, suggesting that delivering an adequate amount of L-citrulline is crucial to ensure vascular benefits. Thus, food technology (e.g., spray and freezing dryer procedures, among others) has been applied to guarantee sufficient dosage or preserve the content of L-citrulline in watermelon products to activate the arginine-NO pathway. Therefore, this narrative review aims to discuss the current evidence on the effects of watermelon ingestion on vascular health parameters and explores the critical relevance of food technologies for adequate L-citrulline content in these products. 

## 2. L-Citrulline Properties

L-citrulline is a neutral non-essential amino acid [13]. The endogenous synthesis of L-citrulline occurs in the enterocytes, where many enzymes convert amino acids derived from the diet to L-citrulline [14]. For example, in the urea cycle (a critical way to detoxify ammonia), the ornithine carbamoyltransferase enzyme converts L-ornithine into L-citrulline [14,15]. In addition, L-glutamine and L-arginine are indirect sources of L-citrulline, as L-arginine can be converted to L-ornithine by the enzyme arginase [16]. Moreover, ornithine aminotransferase utilizes glutamine to produce L-ornithine [16], which can be converted to L-citrulline.

**Figure 1:**
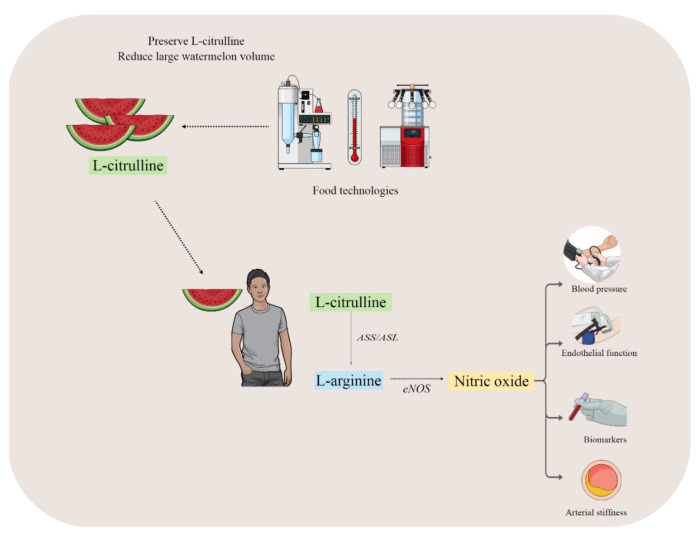
After watermelon ingestion, L-citrulline can be converted into L-arginine by argininosuccinate lyase (ASL) and argininosuccinate synthase (ASS), increasing plasma L-arginine level, which can be converted into nitric oxide (NO) in endothelial cells. Thus, NO can improve vascular health parameters, such as blood pressure, endothelial function, arterial stiffness, and biomarkers. However, due to the large watermelon volume needed to achieve the effective L-citrulline dosage, food technologies are warranted to produce watermelon products with higher L-citrulline content.

L-citrulline can also be produced during NO synthesis. In this pathway, L-arginine is a substrate for endothelial NO synthase (eNOS) enzyme that produces NO and L-citrulline [17]. Interestingly, L-citrulline can be recycled to L-arginine through the action of two enzymes argininosuccinate synthase (ASS) and argininosuccinate lyase (ASL) providing L-arginine for NO production in the endothelium [18,19]. Since L-citrulline is a precursor for L-arginine, studies have evaluated the impact of L-citrulline supplementation on NO bioavailability and vascular function. In addition, previous studies have shown that L-citrulline can be more efficient for increasing plasma L-arginine than L-arginine supplementation per se since unlike L-arginine, L-citrulline is not metabolized by arginase in the intestine, liver, and endothelium [18,19]. In this context, L-citrulline can be an important dietary supplement to increase L-arginine and NO bioavailability and improve vascular function (Figure 1).

L-citrulline can be obtained from the diet and by supplementation. Several studies have utilized synthetic L-citrulline to improve plasma L-arginine and NO bioavailability [2,3,20,21]. In a pharmacokinetic study with different doses of L-citrulline (2, 5, 10, and 15 g), Moinard et al. [22] showed that L-arginine synthesis saturation begins to occur at the highest dose of L-citrulline (15 g), suggesting that lower doses should be adequate for clinical practice.

L-citrulline was first isolated from watermelon (*Citrullus lanatus*), coining the L-citrulline name [13]. Watermelon is the major source of L-citrulline, and several factors can impact its concentration, such as environmental (i.e., exposure to the stress of drought and high light intensity) and physiological aspects (i.e., cultivar, genotype, flesh color and fruit anatomy) [23]. Akashi et al. [24] showed that the central portion of watermelon (pulp) has lower L-citrulline concentration than the peripheral portion (rind). In addition, Rimando and Perkins-Veazie [25] showed variation of L-citrulline content in different flesh colors of watermelon. The red watermelon varieties (Jamboree, Sangria, and Summer Flavor 800) had less L-citrulline than the orange (Tender Sweet Orange) or yellow (Summer Gold) flesh watermelons [25].

Since watermelon is a major dietary source of L-citrulline, several studies have evaluated the effect of its ingestion on plasma L-arginine levels, NO bioavailability, and vascular parameters [1,4,11,26,27,28]. In these studies, watermelon was ingested as juice [4,26], extract [1,11], puree [27], and microencapsulated [28], all of which are natural sources of L-citrulline that could improve vascular health. Although in smaller amounts than L-citrulline, watermelon also contains L-arginine, an amino acid that is directly involved in NO synthesis [29]. As mentioned above, eNOS uses L-arginine as a substrate to produce NO and L-citrulline. Previous studies have demonstrated that L-arginine supplementation can improve cognitive function of hypertensive frail older adults [30] and enhance the effects of cardiac rehabilitation on physical performance of patients who underwent coronary revascularization after acute myocardial infarction [31], since L-arginine exert beneficial effect on endothelium driving vasodilation through NO. Furthermore, L-arginine can improve the immune response since it has been shown that T cell function is dependent on L-Arginine levels [32].

It is important to note that watermelon also contains lycopene, an antioxidant compound that could affect vascular health [33]. However, a previous study showed that watermelon juice ingestion increased plasma lycopene without improvements in vascular function evaluated by flow-mediated dilation and pulse wave velocity in healthy postmenopausal women [9].

## 3. Mechanism of Vascular (Dys) Function

Structural and functional modifications of the arteries are frequently observed in individuals at risk for cardiovascular diseases. The arterial wall has three layers (endothelium, media, and adventitia) responsible for several roles in vascular function [34]. The endothelium is responsible for synthesizing many vasoactive substances, such as NO. The NO molecule plays several roles in the cardiovascular system, including vasodilation, inhibition of smooth muscle cell growth and platelet aggregation, and leukocyte adhesion to endothelial cells [35,36]. Thus, NO availability prevents arterial stiffening, atherosclerosis, and thereby, development of hypertension and cardiovascular diseases. Reduction of NO bioavailability is observed in individuals with several cardiometabolic risk factors and diseases such as hypertension [37], diabetes *mellitus* [38], and hypertriglyceridemia [39].

Reduced NO bioavailability (i.e., reduced NO synthesis and/or increased NO degradation) is the main characteristic of endothelial dysfunction. Increased NO degradation can occur by the activity of several enzymes, such as nicotinamide adenine dinucleotide phosphate (NADPH), NADPH oxidase (NOX), xanthine oxidase, and uncoupled eNOS, which further increases ROS [7]. For instance, NOX catalyzes the NADPH-dependent reduction of oxygen to superoxide anion under pathological conditions. In turn, superoxide anion can react with NO to produce peroxynitrite, and both ROS uncouple eNOS by promoting tetrahydrobiopterin (BH_4_) oxidation to BH_2_ [40].

Uncoupled eNOS has been reported in clinical conditions, such as essential hypertension, diabetes *mellitus*, and hypercholesterolemia [6,7]. The eNOS enzyme is a dimer that contains a bidomain structure, the reductase (C-terminal) and oxygenase (N-terminal) domains [41]. The N-terminal contains BH_4_, heme iron, and L-arginine binding sites. L-arginine and BH_4_ are responsible for stabilizing the active dimeric form of eNOS. For example, eNOS change its heme iron to a high-spin state when BH_4_ is bound to the enzyme, leading to the increases of L-arginine binding [42]. However, under increased oxidative stress conditions (hypertension, diabetes *mellitus*, and hypercholesterolemia), peroxynitrite can oxidase BH_4_ to BH_2_, reducing the BH_4_ levels [43]. The eNOS dimer is uncoupled into two monomers, which generate large amounts of peroxynitrite instead of NO [41,42]. 

In addition to NO degradation, some factors can reduce NO synthesis, including reduced levels of L-arginine [6]. Moreover, in conditions with increased oxidative stress, L-arginine binding to the eNOS can be decreased by competition with asymmetric dimethylarginine (ADMA), an analog of L-arginine and eNOS inhibitor. Oxidative stress reduces the activity of dimethylarginine dimethylaminohydrolase (DDAH), an enzyme responsible for eliminating ADMA, leading to an increase in plasma ADMA.

Reduced NO levels are associated with impaired vasodilation in conduit and resistance vessels [44]. Ghiadoni et al. [44] evaluated flow-mediated dilation (FMD) (a measure of endothelial function in conduit arteries) before and after infusion of N^G^-monomethyl-L-arginine (L-NMMA), an eNOS inhibitor. The authors showed that before infusion of L-NMMA, hypertensive patients showed a significant reduction of FMD compared to normotensive individuals. Furthermore, it was observed that after infusion of L-NMMA, FMD was impaired in normotensive individuals while it was unchanged in hypertensive patients. Collectively, these results demonstrate that reduced NO bioavailability by eNOS inhibition impairs vasodilation in conduit arteries.

Flow-mediated dilation (FMD) is widely used to evaluate endothelial function in conduit arteries, such as brachial and femoral [45,46]. This measure evaluates the increase in artery diameter in response to transient vascular occlusion. During the test, the tissue distal to the occlusion experiments ischemia that results in increased production of metabolites that induce reactive hyperemia after release of the “upstream” occlusion. The robust hyperemia increases laminar shear forces on the endothelium, increasing eNOS activity and NO production [47]. Thus, the percent changes in artery diameter observed in the FMD measurement represent the ability of the endothelium to produce NO dependent vasodilation in response to shear stress [45]. A previous study showed that a 1% increase in FMD was associated with a 9% decrease in future risk of cardiovascular events [45].

In addition to impaired vasodilation, reduced NO bioavailability can cause structural changes in the arterial wall leading to atherosclerosis and arterial stiffening [48]. NO is responsible for inhibiting platelet activation, leukocyte adhesion and migration, and vascular smooth muscle cell (VSMC) proliferation and migration [40]. Endothelial dysfunction has been associated with arterial stiffening [49]. The media layer of elastic arteries has a high content of elastic fibers, which is essential for attenuating the impact of stroke volume on systolic blood pressure and blood flow to the organs [34]. Distensibility of the arterial wall is essential to modulate blood pressure and flow waves throughout the vascular tree [50]. Aortic stiffening associated with aging and cardiometabolic risk factors is the consequence of an increase in collagen fibers, decreased amount of elastin, and abnormal fiber distribution in the arterial wall [48]. Stiffening of elastic arteries (carotid and aorta) can promote systolic hypertension, increased pulsatile hemodynamic load, left ventricular hypertrophy, coronary ischemia, and heart failure [51]. Thus, aortic stiffness is considered an independent risk factor for cardiovascular events [52].

Pulse wave velocity (PWV) has been used to evaluate arterial stiffness [34]. Left ventricle ejection generates a pulse wave that travels forward to the peripheral arterioles [34]. Increased PWV indicates a faster pulse wave propagation in rigid arteries. The arterial segments most used in research are the carotid-femoral PWV (cfPWV) and brachial-ankle PWV (baPWV), which are measures of aortic and systemic PWV, respectively. Studies have shown that cfPWV and baPWV are independent predictors of cardiovascular events and morbidity [53,54].

The augmentation index (AIx) has been used to evaluate the influence of arterial stiffness on wave reflection [55]. The early return of reflected waves from peripheral reflecting sites to the aorta during late systole rather than diastole can increase systolic, pulse pressure and left ventricle afterload. AIx reflects the contribution of wave reflection to the increased aortic pulse pressure [55,56,57]. Some conditions such as aging [58], hypertension [59], diabetes mellitus [60], and hypercholesterolemia can increase AIx. Aortic hemodynamics including systolic pressure and AIx predict cardiovascular events independently of peripheral pressures, indicating the clinical importance of central pressures [61].

## 4. Evidence of Watermelon Ingestion on Vascular Health

### 4.1. Endothelial Function

Several previous studies have evaluated the effect of watermelon products on macro- and microvascular responsiveness assessed by brachial FMD and near-infrared spectroscopy, respectively [5,8,9] (Table 1). Vincellete et al. [8] investigated the effect of a two-week watermelon juice ingestion (500 mL–795 mg of L-citrulline) on attenuating acute hyperglycemia-induced vascular dysfunction in young healthy adults. It was observed that watermelon juice increased the postprandial FMD and microvascular blood flow area under the curve (AUC) (slope of the linear increase in total hemoglobin) compared to placebo. The authors concluded that watermelon could preserve endothelial function, skeletal muscle microvascular oxygen saturation, and blood flow during postprandial hyperglycemia. However, watermelon supplementation did not improve fasted and postprandial FMD%, which is the gold standard measure of conduit artery endothelial function. 

Similarly, ingestion of 360 mL of watermelon juice twice a day (1.63 g of L-citrulline) for four weeks did not improve brachial FMD in healthy postmenopausal women [9]. Moreover, Fan et al. [5] evaluated the acute effect of different portions of watermelon on FMD in healthy overweight and obese subjects. The authors observed that one serving (equivalent to 100 Kcal) of watermelon pulp (10 mg of L-citrulline), watermelon rind (19.3 mg of L-citrulline), or watermelon seeds (1.4 mg of L-citrulline) did not change FMD over 7 h after ingestion. 

In addition to the studies that have evaluated the effect of short-term watermelon ingestion on endothelial function in healthy individuals [5,9,10], Cutrufello et al. [12] reported that acute ingestion of 710 mL of watermelon juice (1 g of L-citrulline) was not efficient for improving brachial artery FMD in healthy active men and women. Overall, these studies found that acute and chronic watermelon supplementation does not improve fasting brachial FMD% in healthy adults. In agreement with findings from watermelon studies, previous studies do not support beneficial effects of L-arginine or L-citrulline supplementation on FMD in healthy individuals [20,62], particularly in young adults. These data indicate that supplementations with NO precursors have no beneficial effect on FMD in individuals with normal endothelial function [63].

### 4.2. Arterial Stiffness and Aortic Hemodynamics

Since NO deficiency plays an essential role in regulating vascular tone and abnormal structural vascular changes, previous studies have evaluated the effect of L-citrulline (an indirect precursor of NO synthesis) on arterial stiffness (Table 2). Figueroa et al. [64] evaluated the effect of six weeks watermelon powder supplementation (containing 2.7 g of L-citrulline and 1.3 g of L-arginine) on aortic stiffness (cfPWV) in individuals with prehypertension. It was demonstrated that watermelon powder did not reduce aortic stiffness in middle-aged individuals with prehypertension. Similarly, Ellis et al. [9] evaluated the effect of 360 mL watermelon juice ingestion twice a day (1.63 g of L-citrulline) for four weeks, and it was observed that watermelon juice did not modify calculated aortic stiffness in healthy postmenopausal women. The lack of effect of watermelon supplementation on arterial stiffness observed in Ellis et al.’s [9] study can be explained by the lower dose of L-citrulline (i.e., 1.63 g of L-citrulline daily) and good health of the participants. For example, an increased plasma L-citrulline and L-arginine after watermelon supplementation was not observed differently in previous studies that have used a dosage of 2 g or even higher [26,27,65].

Previous studies that observed an improved arterial stiffness after ingestion of watermelon products used > 2.7 g of L-citrulline daily, suggesting that a higher L-citrulline dosage is necessary to improve arterial stiffness. Ochiai et al. [66] showed that supplementation with 5.6 g of L-citrulline for seven days reduced baPWV of healthy adults. Thereafter, Figueroa et al. [2] demonstrated that watermelon extract (4 g and 2 g of L-citrulline and L-arginine) ingestion for six weeks reduced baPWV in obese postmenopausal women with hypertension. These findings demonstrated the efficiency of watermelon supplementation to reduce baPWV, a measure that includes peripheral and central arterial stiffness. Since watermelon supplementation reduces baPWV but not cfPWV [66], the benefit may be localized to peripheral arteries, but not the aorta.

Arterial stiffness increases wave reflection from peripheral arteries back to the aorta. Watermelon powder reduced AIx and AIx normalized for a heart rate of 75 beats/min (AIx75), suggesting a reduced left ventricle afterload and risk for cardiovascular events in individuals with prehypertension [1]. Evidence has demonstrated that vascular benefits of L-CIT supplementation, if not apparent at rest, can be evident during sympathetic stimulation induced during the cold pressor test (CPT) in older adults [67]. Figueroa et al. [10] investigated the effect of watermelon extract (4 g of L-citrulline) for 6 weeks on aortic hemodynamic responses to CPT in middle-aged adults with hypertension. It was observed that watermelon supplementation lowered the magnitude of augmented pressure response to CPT. These results suggest that watermelon attenuated cold-induced aortic hemodynamic responses. 

### 4.3. Blood Pressure

The beneficial effect of watermelon ingestion has been observed on blood pressure. Studies have demonstrated that watermelon can significantly lower levels of both systolic (SBP) and diastolic blood pressure (DBP) [2,9] (Table 3). Figueroa et al. [10] showed that ingestion of watermelon extract (4 g of L-citrulline) for six weeks reduced resting brachial and aortic SBP and DBP in middle-aged adults with hypertension and obesity. In addition, ingestion of watermelon extract (4 g and 2 g of L-citrulline and L-arginine) for six weeks lowered aortic SBP and DBP in postmenopausal women [2]. Additionally, Massa et al. [11] demonstrated that watermelon extract (4 g of L-citrulline) supplementation for six weeks reduced brachial SBP and DBP in prehypertensive and hypertensive individuals. In contrast, watermelon juice supplementation did not affect resting blood pressure in healthy postmenopausal women [9]. Furthermore, watermelon snack ingestion for four weeks did not reduce blood pressure in overweight and obese adults [68]. These findings suggest that supplementation with watermelon with at least 4 g of L-citrulline seems effective in reducing blood pressure in individuals with elevated blood pressure and hypertension, but not in normotensives.

### 4.4. Vascular Biomarkers

In the presence of cardiometabolic risk factors (i.e., hypertension, obesity, prediabetes, and hypercholesterolemia) occurs endothelial cell activation, which is characterized by the expression of cell surfaces adhesion molecule, such as vascular cell adhesion protein 1 (VCAM-1), intercellular adhesion molecule-1 (ICAM-1), and E-selectin. Proinflammatory cytokines induce this process as interleukin-1 (IL-1) and tumor necrosis factor (TNF-α) are secreted in response to interleukin-6 (IL-6). With the development of endothelial dysfunction induced by these molecules, the arteries become susceptible to the atherosclerosis process [69]. These cell surface adhesion molecules are widely used as a biomarker of endothelial dysfunction [70].

Although previous studies have demonstrated a positive effect of watermelon ingestion on arterial stiffness, AIx [2,10,65], and blood pressure [2,10,11], previous studies have not observed a significant effect of watermelon on vascular function and biomarkers [11,27,68] (Table 4). Ingestion of 2 cups of fresh diced watermelon for four weeks did not change plasma C-reactive protein (CRP) in overweight and obese adults [65]. Ellis et al. [9] observed that plasma ADMA was unaffected by ingestion of 360 mL watermelon juice twice a day (1.63 g of L-citrulline) for four weeks in healthy postmenopausal women. Two recent studies [8,9] did not observe significant increases in circulating L-arginine following watermelon supplementation, indicating that insufficient substrate for NO production may have contributed to the lack of improvement in FMD. Moreover, Shanely et al. [27] demonstrated that 710 mL of watermelon puree (2.28 g of L-citrulline/L-arginine) for six weeks reduced sVCAM-1, but not soluble platelet selectin (sP-Selectin), high-sensitivity C-reactive protein (hs-CRP), and soluble intercellular adhesion molecule-1 (sICAM-1) in overweight and obese postmenopausal women. 

In contrast, previous studies have observed a decrease in biomarkers of vascular dysfunction with synthetic L-citrulline supplementation [66,71,72]. Supplementation with 5.6 g of L-citrulline the L-arginine/ADMA ratio increased in healthy middle-aged men [66]. Furthermore, Schwedhelm et al. [20] demonstrated that 3.2 g and 6 g of L-citrulline increased plasma L-arginine level and arginine/ADMA ratio in healthy adults. Increased arginine/ADMA ratio favors L-arginine binding to eNOS for NO production. In addition, the supplementation with 3 g of L-citrulline for eight weeks reduced TNF-α and hs-CRP levels in patients with type 2 diabetes *mellitus* [72]. Furthermore, Darabi et al. [71] showed that L-citrulline supplementation (2 g daily for twelve weeks) reduced NF-κb, TNF-α, and Hs-CRP levels in patients with non-alcoholic fatty liver disease. These data suggests that L-citrulline reduces chronic inflammation, a mechanism of endothelial dysfunction. However, it is important to note that these studies used higher L-citrulline dosages (2–6 g of L-citrulline) than studies supplementing with watermelon products (~1.88 g of L-citrulline), which could partially explain the divergent findings.

## 5. Food Technology

Current evidence shows that watermelon product ingestion can be an important strategy to improve vascular function since it is a food source of L-citrulline and L-arginine, amino acids indirectly and directly involved in NO synthesis [18,19]. However, the amount of L-citrulline present in watermelon may be a limiting factor for its beneficial effect on vascular function.

Studies have used a higher quantity of watermelon with a low L-citrulline content. For example, in the Vincellete et al. [12] study, participants ingested 500 mL of watermelon juice to achieve 795 mg of L-citrulline. In addition, Shanely et al. [27] offered 710 mL of watermelon puree containing 1.88 g of L-citrulline. Ellis et al. [9] used 720 mL of watermelon juice containing 1.63 g of L-citrulline. Although a great volume of watermelon juice or puree was provided, the supplementation did not improve vascular function [9,27]. Increased plasma arginine is important for improving endothelial function and watermelon supplementations failed to provide this effect [8,9]. Thus, it is likely necessary to provide a higher volume of watermelon juice or puree (>720 mL) and L-citrulline content to improve vascular function. 

To reduce the large volume of watermelon, some studies have used powder [2,10] to achieve adequate L-citrulline content. The authors used watermelon powder containing 4 g and 2 g of L-citrulline and L-arginine, equivalent to ~2.3 pounds (~1 kg) of raw red watermelon. The powder consisted of sieved and freeze-dried watermelon extract. In this way, a proper L-citrulline dose could be achieved in a lower watermelon quantity.

In addition to the freeze-drying process, researchers have used the microencapsulation technique to concentrate and protect nutrients [28,73,74]. The spray drying process using hot air promotes the atomization of a solution (e.g., fruit juice) into a solid concentrated powder [75]. In a recent study, the authors achieved 4 g of L-citrulline in 30 g of microencapsulated watermelon rind, which effectively increased plasma L-citrulline and L-arginine in healthy adults. Thus, microencapsulation provides sufficient L-citrulline content in a lower watermelon volume to promote potential beneficial vascular effects [28]. 

Moreover, microencapsulation of watermelon using a spray dryer protects and preserves L-citrulline content in food [74]. For example, it has been demonstrated that L-citrulline content can be reduced in watermelon juice when stored at room temperature and 4 °C [74], which could negatively impact the beneficial effect of L-citrulline on vascular function. On the other hand, microencapsulated watermelon exhibits higher L-citrulline stability at these temperatures, demonstrating that microencapsulation can preserve L-citrulline content [74].

Watermelon juice is an extremely attractive product for consumers due to its high nutritional value and sensory properties. For this reason, adequate technologies to help maintain the bioactive compounds and the sensorial quality of the watermelon juice are warranted. The food industry widely applies thermal treatment to preserve fruit juice. However, this food technology can reduce bioactive compounds and modify sensory aspects of watermelon juice [76]. Thus, studies have investigated the best conditions to preserve functional and sensory parameters in watermelon juice. For example, Tarazona-Díaz et al. [77] evaluated different pasteurization treatments (80 °C, for 40 s or 90 s) for the shelf life of watermelon juice. The authors demonstrated that watermelon juice pasteurized at 80 °C for 40 s has higher shelf life than 90 s. However, both conditions reduced L-citrulline content by ~19%. Thus, although thermal treatment is necessary for preserving watermelon, it can reduce the L-citrulline content.

Due to L-citrulline losses during the thermal process of watermelon juice, non-thermal technologies have been evaluated to preserve L-citrulline content in watermelon juice, such as high-pressure processing (HPP) and forward osmosis (FO) [78]. Milczarek et al. [78] investigated the effect of FO on the L-citrulline content of watermelon juice since FO is capable of concentrating liquid at ambient temperature [79]. However, the authors observed no difference in L-citrulline content between FO and other technologies (i.e., HPP, thermal processing, fresh juice). 

Developing new food products with high nutritional value is also a concern of the food industry. For this reason, studies have added watermelon as a nutritional ingredient in food preparations to increase the nutritional value [80,81]. For example, Sadji et al. [81] added 5% of watermelon puree into a bread mixture (wheat flour, salt, yeast, water, and bread improver) and observed several technological and nutritional advantages. Although the L-citrulline content in the bread mixture (i.e., 30 mg L-citrulline) may be too low to acutely promote vascular benefits, improvements in the bread-making process using watermelon puree could be a new approach to increase daily ingestion of L-citrulline.

In summary, food technology is essential to guarantee enough L-citrulline content in watermelon products. These technologies could be useful to reduce the large watermelon volume ingestion and improve adherence to the supplementation protocol [28,74]. In addition, these technologies can reduce L-citrulline losses in watermelon juice [77]. Furthermore, using watermelon to develop new food products could be a strategy to increase daily L-citrulline ingestion.

## 6. Conclusions

Watermelon products (juice, extract, powder, puree) ingestion can lower blood pressure and arterial stiffness when containing sufficient L-citrulline, particularly in clinical populations with cardiometabolic risk factors. Regarding endothelial function, it seems that watermelon intake does not affect this parameter in healthy adults. However, evidence is scarce, and studies have provided very low L-citrulline dosages using watermelon juice. Applying food technologies (spray dryer, freeze-drying, among others) to increase and preserve L-citrulline content in a reduced volume of watermelon is warranted. Watermelon microencapsulation could be a more convenient and effective method for improving adherence and vascular health in individuals with cardiometabolic risk factors.

## Figures and Tables

**Table 1:** Summary of the studies that evaluated the effect of watermelon ingestion on endothelial function.

Study	Population	Intervention	Measure	Outcomes
Vincellette et al. [8]	*n* = 17 (6M/11F) Healthy young adults db, r, crossover	500 mL of watermelon juice (795 mg of L-citrulline) for two weeks	FMD NIRS	↔ FMD (%) ↑ FMD AUC (%. min) ↑ Blood flow AUC ↑ Peak O_2_ total AUC
Ellis et al. [9]	*n* = 17 Healthy postmenopausal women db, r, crossover	360 mL of watermelon juice twice a day for four weeks (1.63 g of L-citrulline)	FMD	↔ FMD (%)
Fan et al. [5]	*n* = 6 Overweight/obese subjects db, r, crossover	Watermelon rind (19.3 mg of L-citrulline) or watermelon flesh (10 mg of L-citrulline) or watermelon seeds (1.4 g of L-citrulline) 1, 3, 5, and 7h prior the analysis	FMD	↔ FMD (%)
Cutrufello et al. [12]	*n* = 22 (11M/11F) Healthy adults db, r, crossover	710 mL of watermelon juice (1 g of L-citrulline)	FMD	↔ FMD (%)

↑ = statistically significant increase; ↔ = no effect; AUC = area under curve; db = double-blind; F = female; FMD = flow-mediated dilation; M = male; NIRS = near-infrared spectroscopy; O_2_ = oxygen; r = randomized; SmO_2_Peak = Highest SmO_2_ value achieved; SmO_2_ = muscle oxygen saturation; SmO_2_RecSlope = muscle oxygen resaturation rate.

**Table 2:** Summary of the studies that evaluated the effect of watermelon ingestion on arterial stiffness and pressure wave reflection.

Study	Population	Intervention	Technique	Outcomes
Figueroa et al. [64]	*n* = 9 (4M/5F) Middle-aged individual’s with prehypertension. db, r, crossover	Watermelon powder (2.7 g of L-citrulline) for six weeks	Tonometry	↓ AIx (%) ↓ AIx75 (%) ↔ cfPWV (m/s)
Ellis et al. [9]	*n* = 17 Postmenopausal women db, r, crossover	360 mL of watermelon juice (1.63 g of L-citrulline) for four weeks	Mobil-O-Graph system	↔ calculated aPWV (m/s)
Figueroa et al. [2]	*n* = 12 postmenopausal women r, crossover	Watermelon extract (4 g of L-citrulline) for six weeks	Tonometry	↓ baPWV (m/s)↔ aAIx (%) ↔ rAIx (%)
Figueroa et al. [10]	*n* = 13 (3M/10F) Middle-aged adults’ with hypertension, obesity, and sedentary db, r, crossover	Watermelon extract (4 g of L-citrulline) for six weeks	Tonometry rest and during cold pressor test	↔ AIx (%) ↔ AIx75 (%) ↓ ∆AIx75 (%)

↓ = statistically significant reduce; ↔ = no effect; aAI_x_ = aortic augmentation index; AI_x@_75 = AIx adjusted for 75 beats/min; baPWV = brachial-ankle PWV; cfPWV = carotid–femoral pulse wave velocity; db = double-blind; F = female; faPWV = femoral-ankle PWV; M = male; PWV = pulse wave velocity; rAI_x_ = radial augmentation index; r = randomized.

**Table 3:** Summary of the studies that evaluated the effect of watermelon ingestion on blood pressure.

Study	Population	Intervention	Outcomes
Figueroa et al. [10]	*n* = 13 (3M/10F) Middle-aged adults with hypertension, obesity, and sedentary db, r, crossover	Watermelon extract (4 g of L-citrulline) for six weeks	↓ aSBP (mmHg) ↓ aDBP (mmHg) ↓ bSBP (mmHg) ↓ bDBP (mmHg)
Figueroa et al. [2]	*n* = 12 Postmenopausal women r, crossover	6 g of watermelon extract (4 g of L-citrulline) for six weeks	↓ aSBP (mmHg) ↓ aDBP (mmHg)
Massa et al. [11]	*n* = 40 Prehypertensive and hypertensive individuals db, r, crossover	6 g of watermelon extract (4 g of L-citrulline) for six weeks	↓ bSBP (mmHg) ↓ bDBP (mmHg)
Ellis et al. [9]	Postmenopausal women (*n* = 17) Db, r, crossover	360 mL of WJ twice a day for four weeks (360 mL WJ = 1.63 g of L-citrulline)	↔ Office SBP (mmHg) ↔ Office DBP (mmHg) ↔ Office Pulse Pressure (mmHg) ↔ Office Pulse Pressure Amplification ↔ 24-Hour ABPM SBP (mmHg) ↔ 24-Hour ABPM DBP (mmHg)
Lum et al. [68]	*n* = 23 (20M/13F) Overweight and obese adultscrossover	2 cups of fresh watermelon for four weeks	↔ bSBP (mmHg) ↔ bDBP (mmHg) (only in men)
Figueroa et al. [66]	*n* = 9 (4M/5F) Middle-aged individuals with prehypertension. db, r, crossover	Watermelon powder (2.7 g of L-citrulline) for six weeks	↔ bSBP (mmHg) ↔ bDBP (mmHg) ↓ aSBP (mmHg) ↔ aDBP (mmHg)

↓ = statistically significant reduce; ↔ = no effect; ABPM, ambulatory blood pressure; aDBP, aortic diastolic blood pressure; aSBP, aortic systolic blood pressure; bDBP, brachial diastolic blood pressure; bSBP, brachial systolic blood pressure; db = double-blind; M = male; F = female; r = randomized.

**Table 4:** Summary of the studies that evaluated the effect of watermelon ingestion on plasma biomarkers.

Study	Population	Intervention	Outcomes
Lum et al. [68]	*n* = 23 (20M/13F) Overweight and obese adults Crossover	2 cups of fresh watermelon for four weeks	↔ CRP (mg/L)
Ellis et al. [9]	Postmenopausal women (*n* = 17) db, r, crossover	360 mL of WJ twice a day for four weeks (360 mL WJ = 1.63 g of L-citrulline)	↔ ADMA (µM)
Shanely et al. [27]	*n* = 51 Overweight and obese postmenopausal women r, parallel	710 mL of watermelon puree (1.88 g of L-citrulline) for six weeks	↔ sVCAM-1 (ng/mL) ↔ sP-Selectin (ng/mL) ↔ hs-CRP (mg/L) ↔ sICAM-1 (ng/mL)

↔ = no effect; ADMA, asymmetric dimethylarginine; CRP, C-Reactive Protein; db = double-blind; F = female; hs-CRP, high-sensitivity C-reactive protein; M = male; r = randomized; sICAM-1 = soluble intercellular adhesion molecule-1; sP-Selectin = soluble platelet selectin.

## Data Availability

Not applicable.
